# Der Einfluss von IoT-, Big-Data- und Mobile-Health-Lösungen auf die Wertschöpfung in Krankenhäusern: Gap-Analyse und Handlungsempfehlungen

**DOI:** 10.1365/s40702-020-00682-z

**Published:** 2020-11-11

**Authors:** Florian Neft, Karolin Eva Kappler, Stefan Smolnik

**Affiliations:** FernUniversität in Hagen, Hagen, Deutschland

**Keywords:** Digitalisierung, Nachhaltigkeit, Gesundheitswesen, Wertschöpfung, Technologie, COVID-19, Digitization, Sustainability, Healthcare, Value chain, Technology, COVID-19

## Abstract

Deutsche Krankenhäuser sehen sich heutzutage mit Herausforderungen wie der Globalisierung, dem demographischen Wandel, nachhaltiger Entwicklung und den Folgen des Corona-Virus konfrontiert. Um in diesen Zeiten die Versorgungsqualität zu verbessern, profitabel zu wirtschaften und die Resilienz gegenüber diesen allgemeinen und spezifischen Risiken zu erhöhen, ist in Krankenhäusern ein Optimierungsbedarf erkennbar. Daher werden in dieser Arbeit Mängel im Leistungsspektrum deutscher Krankenhäuser untersucht, um darauf aufbauend aktuelle Lösungsansätze zu diskutieren. Die hierfür interviewten Krankenhausleiter erkennen in der Kommunikation, der Reaktionsfähigkeit bei Patientenanstiegen, der Verwaltung sowie der IT-Ausstattung Verbesserungspotenziale. Die Antworten zu den verwendeten IT-Systemen weisen darauf hin, dass die Krankenhäuser bereits diverse Technologien einsetzen, diese allerdings häufig veraltet sind, zu wenige Schnittstellen besitzen und somit die Anforderungen des Personals und der Patienten nicht erfüllen. In der Evaluierung zeigen sich durch die Verwendung von Informations- und Kommunikationstechnologien wie Mobile Health, Big Data und dem Internet of Things langfristig Potenziale, die Leistungs- und Nachhaltigkeitsdefizite zu lösen. Weiterhin tragen sie zur Verbesserung vieler Wertschöpfungsprozesse bei. Auch für andere Technologien wie Robotik, Virtual und Augmented Reality sowie RFID bestehen zahlreiche Nutzungspotenziale. Dennoch bindet die Digitalisierung personelle und finanzielle Mittel, dient aber durch verbesserte Planungsmöglichkeiten der Ressourceneinsparung und somit der Nachhaltigkeit.

## Abstract (en)

Globalization, demographic change, sustainable development, and implications of the Corona virus challenge German hospitals today. In order to improve the quality of care, to operate profitably, and to increase resilience to these general and specific risks, hospitals need to optimize. For this reason, we examine deficiencies in the services offered by German hospitals, in order to discuss current solutions. The hospital managers interviewed for this purpose identify potentials for improvement in communication, responsiveness to patient increases, administration, and IT equipment. The responses about the IT systems in use indicate that the hospitals already apply various technologies, but that these are mostly outdated, have too few interfaces, and, thus, do not meet the requirements of staff and patients. The evaluation shows the long-term potential of using information and communication technologies such as Mobile Health, Big Data, and Internet of Things to solve deficits concerning performance and sustainability. They also contribute to the improvement of many value-added processes. Promising application areas also exist for other technologies such as robotics, virtual and augmented reality, and RFID. Nevertheless, digitization ties up human and financial resources, but serves to save resources through improved planning and hence to promote sustainability.

## Herausforderungen deutscher Krankenhäuser

Der technische Fortschritt, die erhöhte Personalbelastung sowie die Krisenbewältigung sind Herausforderungen, denen Krankenhäuser heutzutage gegenüberstehen. Der Gedanke einer nachhaltigen Entwicklung führt zudem dazu, dass ökologische, ökonomische sowie soziale Aspekte auch in Krankenhäusern berücksichtigt werden müssen. Aufgrund zunehmender Konkurrenz steigt der Druck auf das einzelne Krankenhaus, während die veränderte Bevölkerungszusammensetzung in Deutschland einen Fachkräftemangel erzeugt. Die fallende Geburtenrate bei gleichzeitig wachsender Lebenserwartung trägt dazu bei, dass die Anzahl des verfügbaren Personals bei steigendem Patientenaufkommen sinkt. Folglich sehen sich deutsche Krankenhäuser mit einer zunehmenden Nachfrage nach Gesundheitsdienstleistungen bei stagnierenden personellen Ressourcen konfrontiert (Porter und Guth 2012). Diese Nachfrage wird zusätzlich durch Viruserkrankungen wie Corona kurzfristig erhöht. Daraus geht eine ungenügende medizinische Behandlung für die Patienten hervor, bei denen Hilfsmittel sowie Zeit fehlen. Aus diesem Grund sollen medizinische Einrichtungen nach Optimierung streben. Im Sinne der Nachhaltigkeit werden hierbei Effizienz (die ergiebigere Nutzung von Materialien und Energie), Suffizienz (die Einsparung von Ressourcen) sowie Konsistenz (alternative Leistungserbringung durch beispielsweise wiederverwendbare Materialien) verfolgt (Jacob 2019). Einen signifikanten Beitrag hierzu kann die Implementierung neuer Technologien im Krankenhaus leisten – es entsteht das „Krankenhaus 4.0“. Der ursprüngliche Begriff „Industrie 4.0“ betrachtet industrielle Lösungen der Digitalisierung. Im Gegensatz zum industriellen Sektor ist das Gesundheitswesen in der Umsetzung solcher Ansätze nicht gleichermaßen fortschrittlich (Porter und Guth 2012). Werden diese neuen Erkenntnisse zu den Informations- und Kommunikationstechnologien (IKT) auf das Krankenhaus übertragen, ermöglichen sie medizinische Leistungen zu verbessern, die Wertschöpfung zu erhöhen sowie Nachhaltigkeitskonzepte umzusetzen. Um Potenziale digitaler Lösungen durch die Nutzung neuer IKT in klinischen Einrichtungen aufzudecken, hilft die Adaption in fortschrittlicheren Industrien wie der Logistik umgesetzter Konzepte. Die Logistik ist aufgrund des Dienstleistungscharakters mit der Gesundheitswirtschaft vergleichbar, weshalb deren Lösungen grundsätzlich auf Krankenhäuser übertragbar sind (Reddy und Brahm 2016). Daher wird in diesem Artikel anhand einer empirischen Erhebung aufgezeigt, inwiefern die vermuteten Leistungs- und Nachhaltigkeitsdefizite in Krankenhäusern existieren und wie diesen mit ausgewählten IKT begegnet werden kann.

## Grundlagen zum Krankenhausmanagement

Ein Krankenhaus dient zur Identifikation und Heilung von häufig schwerwiegenden Krankheiten. Dabei ist jeder Patient spezifisch zu betrachten und ähnliche Diagnosen können differenziert therapiert werden (Bormann 2012). Neben dieser Vielfältigkeit in der Behandlung unterscheiden sich Krankenhäuser hinsichtlich ihrer Organisationsform und Verwaltung, die von einem kommunalen, kirchlichen oder privaten Träger übernommen wird (Bormann 2012). Unabhängig davon werden Krankenhäuser als Kern des Gesundheitssystems angesehen, wobei der Patient im Fokus des Handelns steht (Zapp 2014).

### Leistungserstellung in Krankenhäusern

Krankenhäuser gelten als Symbiose aus Dienstleistungs- und Non-Profit-Organisation. Einerseits erfüllen sie Merkmale wie Immaterialität eines Dienstleistungsunternehmens. Andererseits orientieren sie sich an karitativen Werten, was den Non-Profit Charakter widerspiegelt (Zapp 2014). Auch die Erlösstruktur ähnelt einem Dienstleistungsunternehmen, denn der laufende Betrieb und damit die Behandlungskosten werden von den Krankenkassen innerhalb des „Diagnosis Related Group Modells“ übernommen (Porter und Guth 2012). Dabei wird je Krankheit, unabhängig von der Behandlungszeit, eine Fallpauschale vergütet. Geht die tatsächliche Aufenthaltsdauer aufgrund von Komplikationen über den kalkulierten Zeitraum hinaus, so werden zusätzliche Kosten nicht getragen und Verluste erwirtschaftet (Ohm 2017). Um daher als medizinische Organisation am Markt zu bestehen, muss eine Strategie etabliert werden, die den Leistungserbringer unterstützt, den Patienten in den Mittelpunkt rückt und zur Wertschöpfung beiträgt (Porter und Teisberg 2006). Da die Patienten sowie das Krankenhaus von der bestmöglichen Diagnose sowie Therapie profitieren, stehen diese wertschöpfenden Prozesse in der von Porter und Teisberg (2006) konzipierten „Healthcare Delivery Value Chain“ im Fokus. Kriegel (2012) leitet aus dieser, für das gesamte Gesundheitssystem geltenden Theorie, eine für das Krankenhaus spezifische Prozesskette ab.

**Abb. 1:**
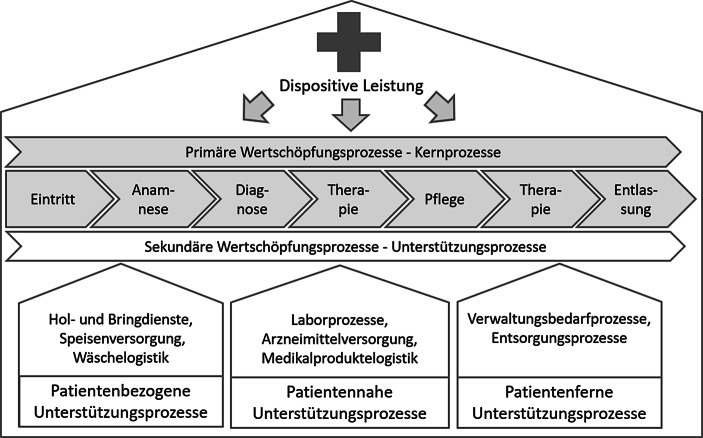
Wertschöpfung im Krankenhaus (angelehnt an Kriegel 2012, S. 84)

Abb. 1 trennt die primären (grau dargestellt) von den sekundären (weiß gekennzeichnet) Prozessen. Erstere konzentrieren sich auf Tätigkeiten, die direkte Auswirkungen auf die Genesung des Patienten besitzen, wie u. a. die Diagnose oder Therapie. Die Primärprozesse werden von patientenbezogenen, -nahen und -fernen Hilfsprozessen unterstützt. Diese sekundären Prozesse wirken sich nicht unmittelbar auf den Heilungsverlauf aus, besitzen aber indirekt Einfluss auf den Patienten, da sie zu dessen Beurteilung der Behandlungsqualität beitragen (Kriegel 2012). Ziel der gesamten Wertstromkette ist gemäß Porter et al. (2016) die erhöhte Wertschöpfung. Fehlt der Fokus auf sie und damit auf die Behandlungsqualität, werden vermehrt Verluste verzeichnet. Derzeit sind 40 % aller deutschen Krankenhäuser insolvenzbedroht (Ohm 2017). Die erste These lautet folglich:

#### T1

*Deutsche Krankenhäuser besitzen Defizite in der Leistungserstellung*.

### Branchenübergreifende Technologien

Gemäß der ersten These erhalten die Patienten eine andere Leistung als erwartet, weshalb sie unzufrieden sind. Ursächlich dafür sind z. B. kürzere Verweildauern, welche die Umsetzung etablierter Behandlungskonzepte nicht mehr zulassen (Bormann 2012). Daher müssen diese mit Blick auf die Patienten sowie die Wertschöpfung erneuert werden. Als Grundlage dafür können branchenähnliche Lösungen herangezogen werden. Aufgrund des Dienstleistungscharakters ist das Krankenhausmanagement mit der Logistik vergleichbar. Dort stiegen durch die Globalisierung die Kundenanforderungen, zugleich sorgten aber auch neue IKT für Vorteile wie z. B. erhöhte Transparenz. Ziel hierbei ist der maximale Kundennutzen. Elementares Merkmal sind die durch den technischen Fortschritt verfügbaren Technologien, die zu positiven Effekten wie vermiedenen Doppelarbeiten führen (Fandel et al. 2009). Diese Digitalisierungsansätze bieten auch im Krankenhaus Potenziale zur erhöhten Patientenorientierung. Trotz dieser Möglichkeiten fehlt in Krankenhäusern oftmals die für die Implementierung neuer digitaler Lösungen notwendige Infrastruktur, die z. B. einen schnellen Datentransfer zulässt (Porter 2014). Daraus folgt die zweite These:

#### T2

*Deutsche Krankenhäuser besitzen Verbesserungspotenziale in der IKT-Nutzung*.

Dennoch ist nicht jede Innovation vorteilhaft. Neben Wettbewerbsvorteilen können erhöhte Kosten die Folge einer Neuerung sein, sodass die Einführung neuer IKT weniger profitabel ist als die Nutzung etablierter IKT (Porter 2014). Neben der Analyse der derzeitigen Situation, ist die Definition von Zielen von Bedeutung für die erfolgreiche Umsetzung. Ein Krankenhausleiter wird sich hierbei primär am Patienten orientieren, denn dieser ist ausschlaggebend für den Erfolg des Krankenhauses (Bormann 2012). Zudem sind die Mitarbeiter, die zukünftig mit neuen IKT arbeiten, zu berücksichtigen. Bietet eine digitale Lösung Mehrwert für Personal und Patienten, liefert sie einen Beitrag zur Wertschöpfung im Krankenhaus (Porter und Teisberg 2006). Entsprechend folgt die dritte These:

#### T3

*Ausgewählte IKT helfen die Defizite in der Leistungserstellung auszugleichen und die Wertschöpfung im Krankenhaus zu erhöhen*.

## Studienaufbau

Die formulierten Thesen werden anhand empirischer Erkenntnisse geprüft. Zur Datensammlung wird eine Kombination aus einer quantitativen Erhebung mittels eines Onlinefragebogens und aus anschließenden qualitativen Experteninterviews gewählt. Beide Befragungen erfassen Querschnittsdaten im Sommer 2019, um bestehende Defizite zu identifizieren und richten sich an Führungskräfte deutscher Krankenhäuser wie technischen Leitern oder Qualitätsmanagern. Die quantitative Erhebung stützt sich auf eine Stichprobe von 94 Antworten. Inhaltlich werden die drei Thesen, die in Tab. 1 umschrieben sind, behandelt.

**Tab. 1:** Inhalt des Onlinefragebogens

Inhalt des Onlinefragebogens	Bewertung
*T1: Gap-Analyse (orientiert an Wilson et al. *2016*)*	*Abschn. 4*
Gap 1: Patientenerwartung ≙ Wahrnehmungen der Krankenhausleitung	Skala 0–5 Punkte/arith. Mittel
Gap 2: Wahrnehmungen der Krankenhausleitung ≙ Definierte Standards	
Gap 3: Definierte Standards ≙ Handlungen des Personals	
Gap 4: Handlungen des Personals ≙ Kommunikation zum Patienten	
Kunden-Gap: Patientenerwartung ≙ erhaltene Leistung des Patienten	
*T2: Technologiedefizite*	*Abschn. 5*
Derzeitige Nutzung ausgewählter IKT^a^	Kumulation
Hindernisse der derzeitigen Nutzung	Textfeld
*T3: Evaluation der IKT*	*Abschn. 5*
Nutzen ausgewählter IKT für das Krankenhaus und Patienten	Skala 0–5 Punkte/arith. Mittel
Kosten ausgewählter IKT für das Krankenhaus	

^a^Im Rahmen dieser Arbeit werden die IKT „Mobile Applikationen & Wearables“, „Robotik“, „Virtual & Augmented Reality“, „RFID“, „Internet of Things“ und „Big Data“ behandelt

Zunächst wird eine Gap-Analyse durchgeführt. Anschließend werden die Technologiedefizite erörtert, um final den Nutzen sowie die Kosten der IKT zu eruieren, welche die Leistungslücken beheben können. Die quantitativen Daten werden durch qualitative in Form von sieben Experteninterviews ergänzt.^1^ Die Durchführung erfolgt in Form 30-minütiger Telefoninterviews. Darin wird zunächst auf die spezifischen Technologie- und Leistungsdefizite eingegangen. Der Fokus liegt auf den Anwendungsmöglichkeiten der IKT und deren Einfluss auf die Wertschöpfung. Zur Auswertung und Strukturierung der Gespräche dient die qualitative Inhaltsanalyse nach Mayring. Die nachfolgenden Kapitel prüfen die Thesen anhand der empirischen Erkenntnisse.

## Gap-Analyse

In Bezug auf Dienstleistungen wie z. B. die Patientenbehandlung besitzen Management, Personal und Patienten unterschiedliche Wahrnehmungen. Daher wird geprüft, ob die Patientenerwartung an die Behandlung der erhaltenen Leistung entspricht. Anhand von zwölf innerhalb der Gap-Analyse gestellten Fragen werden Abweichungen identifiziert. Sechs dieser Fragen werden unterdurchschnittlich bewertet und weisen folglich auf Handlungspotenziale hin, die in Tab. 2 dargestellt sind.

**Tab. 2:** In der Gap-Analyse identifizierte Leistungsdefizite

Defizit	Onlineumfrage (∅ = 3,41)	Aussagen der Experteninterview
***Aktualität der Ausstattung***	∅ = 3,29	**Infrastrukturelle Mängel:** *„Die ganze bauliche Ausstattung [passt] nicht mehr [so] ganz.“ (Int5)* *„IT-Ausstattung ist überall das Problem.“ (Int7)*
***Interne Kommunikation***	∅ = 2,94	**Keine Akzeptanz des Personals für neue IKT:** *„Einige sind noch in der analogen Welt […] und das macht […] die Vielzahl der Mitarbeiterkommunikationsprozesse schwierig.“ (Int5)*
***Verwaltungsprozesse***	∅ = 3,24	**In der Vergangenheit falscher Fokus der sekundären Prozesse:** *„Die haben sich nie auf Kosten und Prozesse konzentriert, sondern immer sehr beschränkt die einzelnen Probleme abgearbeitet.“ (Int3)*
***Reaktionsfähigkeit auf Patientenanstieg***	∅ = 3,13	**Geringe Flexibilität bei Patientenanstiegen:** *„Ich behaupte mal, dass jede Universitätsklinik maximal drei Schwerverletzte parallel […] adäquat behandeln kann. So sind es bei einem unserer Größe gut 550 Betten, wir werden ein bis zwei Patienten, ich sage mal abends um 22 Uhr, schwerstens polytraumatisierte maximal versorgen können. Mehr geht nicht.“* (*Int1)*
***Externe Kommunikation***	∅ = 3,06	**Fehlende IKT zur besseren Kommunikation:** *„Kliniken, wie wir, haben fast 2000 Betten. Dafür gibt es noch zu wenig IT-Systeme, die wirklich die Kommunikation perfekt machen lassen.“ (Int4)*
***→ Kunden-Gap***	∅ = 2,89	–

Die Defizite scheinen u. a. von der Krankenhausgröße abhängig zu sein, da im Kontext der Reaktionsfähigkeit auf erhöhte Patientenzahlen große Krankenhäuser kurzfristig mehr Kranke aufnehmen können, während dies bei einem mittleren Krankenhaus wie in Int1 (siehe Tab. 2) erwähnt nicht der Fall ist. Zusammengefasst zeigen sich Defizite in der Kommunikation, der Reaktionsfähigkeit beim Patientenanstieg, der Infrastruktur und der Verwaltung. In der Summe tragen diese Defizite zu der vom Patienten empfundenen Abweichung zwischen erwarteter und erhaltener Leistung – dem Kunden-Gap – bei. Daher wird die These 1 „*Deutsche Krankenhäuser besitzen Defizite in der Leistungserstellung*“ bestätigt.

## Einfluss ausgewählter IKT auf Defizite und Wertschöpfung

Um die identifizierten Leistungs- und Nachhaltigkeitsdefizite auszugleichen, können analog zur Logistik neue IKT beitragen. Daher wird zunächst die derzeitige Verwendung der IKT reflektiert. Heute arbeiten 90 % der befragten Krankenhäuser mit neuen IKT. Die höchste Nutzungsrate weisen Internet-of-Things-Lösungen (IoT-Lösungen) (64 %) auf. Im klinischen Kontext wird darunter die Verbindung von Diagnostikgeräten mit dem Krankenhausinformationssystem (KIS) verstanden, wobei Laborbefunde via WLAN in die Patientenakte importiert werden (vgl. Int7). Daneben setzen 50 % aller Onlinebefragten Mobile-Health-Systeme ein. Sie erweitern die dargestellte Funktion um mobile Zugriffsoptionen (vgl. Int6). Zudem nutzen 51 % der Krankenhäuser aus der Onlineumfrage Big-Data-Lösungen. Die weiteren in der Onlineumfrage behandelten IKT werden seltener eingesetzt. Die insgesamt verbreitete Verwendung der IKT bedingt allerdings nicht deren gleichzeitig fortschrittlichen und nachhaltigen Einsatz (vgl. Int3). So bezeichnen 54 % der Onlineteilnehmer die bestehende IT-Infrastruktur als veraltet. Dieser Aspekt wird durch Int3 angesprochen:

*Da zeichnet sich das Bild, dass das Krankenhaus technologisch schon eher 10 bis 15 Jahre in logistischen und produktionstechnischen Prozessen und auch von der Technik hinterher ist. (Int3)*

Ursächlich dafür ist das fehlende krankenhausinterne Projektmanagement (vgl. Int2). In der Onlineumfrage erkennen zudem 35 % der Krankenhäuser die fehlende Reife und mangelhafte Bedürfniserfüllung der gegenwärtigen IKT als Hindernis. Diese Aspekte zeigen zudem den fehlenden, derzeit aber immer relevanter werdenden Nachhaltigkeitsgedanken. In Summe wird daher die These 2 „*Deutsche Krankenhäuser besitzen Verbesserungspotenziale in der IKT-Nutzung*“ bestätigt.

**Abb. 2:**
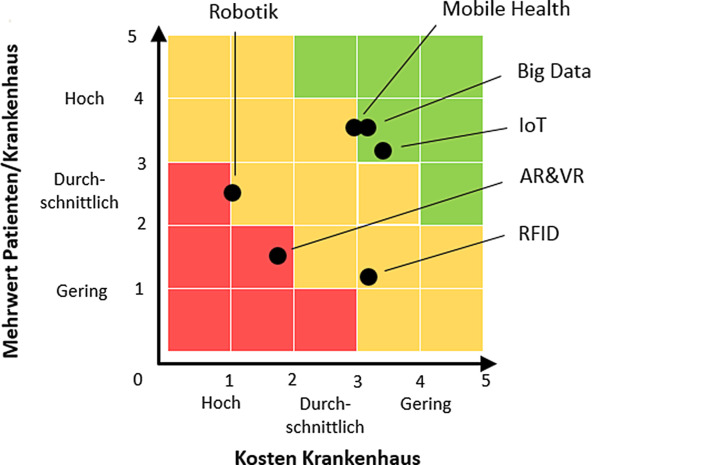
Kosten-Nutzen-Betrachtung der IKT

Folglich werden digitale Lösungen gesucht, die zur Verbesserung der identifizierten Leistungsdefizite führen sowie eine nachhaltige Wirkung bei der Betrachtung von Konsistenz, Effizienz und Suffizienz hervorbringen. (vgl. Int4). Derzeit wollen 91 % der Krankenhäuser mit dem Einsatz neuer IKT die Behandlungsqualität verbessern, 55 % die Therapiezeit verkürzen und 52 % einen Mehrwert für die Patienten schaffen. Diese Ziele stehen in Bezug zur Wertschöpfung, die Kosten und Nutzen der jeweiligen Innovation abwägt. Abb. 2 stellt die Ergebnisse der Onlineumfrage hinsichtlich der Kriterien „Kosten für das Krankenhaus“ sowie „Mehrwert für Patienten und Krankenhaus“ dar.

In Abb. 2 befinden sich zwei Lösungen im grünen, drei im gelben sowie eine im roten Bereich. Big Data, IoT sowie Mobile Health (grüner, oberer gelber Bereich) zeigen Potenziale, weshalb sie im Folgenden detaillierter erläutert werden. Die übrigen IKT (Robotik, AR&VR und RFID) weisen Hindernisse in Form hoher Kosten oder geringen Nutzens auf, sodass auf sie nicht näher eingegangen wird.

### Internet of Things

65 % der Krankenhäuser geben an, dass IoT-Ansätze im Krankenhaus verwendbar sind. In den Experteninterviews wird darunter die Verbindung von Visitenwagen, Diagnostikgeräten oder dem Labor mit dem KIS verstanden. Der Zugriff auf Informationen kann dabei über einen stationären Computer (vgl. Int7) oder mithilfe eines mobilen Endgeräts erfolgen (vgl. Int6). So verwendet ein Krankenhaus iPads, auf denen Ärzte Behandlungsaufträge sowie Patientendaten einsehen und die Visite mobil durchführen können. Folglich kann das Personal jederzeit auf die benötigten Informationen zugreifen, sodass Suchzeiten verkürzt werden, was die internen Verwaltungsprozesse und die Reaktionsfähigkeit bei einem Patientenanstieg verbessert (vgl. Int6). Diese optimierte Kommunikationsmöglichkeit hebt auch Int1 hervor:

*Diese Kommunikation, also ich habe Daten überall zur Verfügung, wird durch digitale Prozesse deutlich vereinfacht und verbessert, […] vor allem wirklich auch gut dokumentiert und jeder weiß woran er ist. Eine große Hilfe. (Int1)*

Neben diesem Beitrag zur Effizienz tragen IoT Lösungen zu einem konsistenten Ablauf der Prozesse im Krankenhaus bei. So wird durch die digitale Verfügbarkeit der Informationen beispielsweise Papier eingespart und in der Folge auch Abfall vermieden. Zugleich können notwendige Tätigkeiten angestoßen werden, wenn sie tatsächlich anfallen. Beispielsweise könnte ein mit dem KIS verknüpfter Abfallcontainer auf einer Station die Meldung geben, sobald er voll ist, oder ein Medikamentenbehältnis könnte eine Nachbestellung veranlassen (vgl. Int2). Insbesondere aufgrund der Notwendigkeit der genannten Informationen im gesamten Krankenhaus besitzt IoT somit einen positiven und nachhaltigen Einfluss auf die gesamte Wertschöpfungskette im Krankenhaus. Demzufolge schaffen diese Lösungen eine Krankenhausinfrastruktur, welche weitere IKT wie die Datenanalyse im Rahmen von Big Data begünstigt.

### Big Data

Weitere 64 % der Onlinebefragten geben an, dass Big-Data-Lösungen in Krankenhäusern verwendbar sind. In den Experteninterviews werden darunter Auswertungen des KIS verstanden, mit denen die interne Kommunikation und Reaktionsfähigkeit verbessert wird (vgl. Int3). Da das KIS medizinische, klinische und verwaltende Informationen über die Patienten sowie das Krankenhaus sammelt, lassen sich durch Analysen Krankheits- und Infektionsverläufe besser nachverfolgen:

*Man stellt ja immer fest, gerade wenn auf der Station irgendwelche Infektionen da sind: Da könnte man schon etwas verknüpfen. Wo sind die Patienten im Urlaub, ist es vielleicht zufällig, dass auf der Station auch eine Krankenschwester schon mal Dienst gemacht hat, in dem Land. Und dann gibt es irgendeine Kombination […], das ist die Ursache für die Verbreitung eines Keims. (Int6)*

Sofern diese Informationen verfügbar sind, können daraus Schlüsse für die Anamnese, Diagnose und Therapie gezogen sowie sowohl die Reaktionsfähigkeit als auch die Kommunikation stark verbessert werden (vgl. Int6). In diesem Kontext kann vor allem die künstliche Intelligenz auf Basis analysierter Laborproben und Vergleichsfällen automatisiert und ohne menschliche Eingriffe Befunde erheben (vgl. Int6). Zudem kann sie Handlungsempfehlungen in Operationen geben. Dabei generiert ein entsprechendes Informationssystem anhand biometrischer Daten, Vitalparameter und mithilfe eines hinterlegten Modells automatisiert Vorschläge zum weiteren Vorgehen (vgl. Int6). Damit kann diese IKT den Eintritt, anstehende Operationen sowie die daran angrenzenden Hol- und Bring-Dienste, Entsorgungsprozesse, Wäsche- als auch Medikalproduktelogistik mit prozessualen Informationen steuern (vgl. Int2; Int4; Int6). Sind diese Informationen vor dem Eintreffen des Patienten im Krankenhaus bereits für den Operateur zugänglich, so kann dieser besser planen (vgl. Int4). Zudem kann das Personal besser organisiert und gesteuert werden. So können Fallwägen für Operationen vorbereitet und die Patientenlogistik abgeklärt werden. Damit wird die Durchlaufzeit des Patienten und somit die Effizienz des Krankenhauses verbessert (vgl. Int4). Weiterhin können Medikationspläne und Laborproben auf Wechselwirkungen analysiert werden, womit Big-Data-Lösungen in der Arzneimittelversorgung sowie den Laborprozessen einen Mehrwert bieten (vgl. Int7). Ist zudem die Zuordnung zwischen Patienten und der jeweiligen medikamentösen Behandlung im KIS registriert, so können lediglich einzelne Pillen auf die Station geliefert und infolge nicht benötigte Restbestände in jeder Abteilung vermieden werden (vgl. Int5). Diese Lösung stellt einen suffizienten Ansatz dar, um Medikamente nicht verfallen zu lassen. Besonders der zukünftige Nutzen von künstlicher Intelligenz in Form organsierterer Abläufe und kürzerer Patientenverweilzeiten wird positiv hervorgehoben (vgl. Int4).

### Mobile Health

Für Mobile Health erkennen 64 % der Teilnehmer der Onlineumfrage Einsatzmöglichkeiten in den Krankenhäusern. Mithilfe mobiler Endgeräte können Patientendaten jederzeit eingesehen werden, was die Kommunikation verbessert, da bei der Visite Röntgenbilder leichter visualisierbar sind (vgl. Int6). Zudem kann das Personal auf einem mobilen Endgerät Anweisungen erhalten, was sich positiv auf dessen Organisation und damit die interne Verwaltung auswirkt. So optimiert eine Navigationsfunktion die Hol- und Bring-Dienste (vgl. Int3). Daneben können Ärzte unter Verwendung eines Smartphones auf Diktate zurückgreifen oder im Internet recherchieren, was Anamnese, Diagnose und Therapie erleichtern (vgl. Int6). Eine Unterform – Wearables – vereinfacht in Kombination mit der künstlichen Intelligenz ebenso den Eintritt und die Entlassung, indem die auf einer Smartwatch gemessenen Daten Verletzungen autonom klassifizieren (vgl. Int6). Anhand dieser automatischen Einordnung der Verletzungsschwere wird die Reaktionsfähigkeit beim Patientenanstieg erhöht (vgl. Int6). Zudem diagnostizieren Wearables mittels integrierter Sensoren Unregelmäßigkeiten wie Herzrhythmusstörungen und können selbstständig den Notruf alarmieren (vgl. Int1). Weiterhin lassen sich mit diesen Geräten unabhängig vom Krankenhauspersonal langfristige Messungen durchführen, was nutzenstiftend für die Pflege sowie Therapie ist (vgl. Int5). Durch eine solche Beobachtung von Vitalparametern können Patienten somit eigenständig präventive Maßnahmen ergreifen, wodurch deren Nachfrage nach Dienstleistungen im Krankenhaus vermieden werden kann. Gleichzeitig können durch die kontinuierliche Beobachtung im Krankenhaus Notfälle früher erkannt werden. So können mit dem Einsatz von Mobile-Health-Geräten einerseits Ressourcen eingespart und andererseits effizienter genutzt werden, weshalb nachhaltige Effekte, u. a. im Bereich der Effizienz und Suffizienz, abgeleitet werden können. Ein weiteres Wearable ist die mit Sensoren verknüpfte Kleidung (vgl. Int3). Anhand eines solchen Arztkittels kann der Kontakt mit Viren schnell erkannt und Sicherheitsvorkehrungen getroffen werden wie Int3 erklärt:

*Ein Arzt, der in ein Zimmer geht und sich da irgendein Virus einfängt: Dann könnte sich das Hemd grün oder rot färben und dann einen Alarm auslösen und dann wüsste der sofort, er muss sich desinfizieren und er muss dringend sein T‑Shirt wechseln und er muss das auch separat abwerfen, sodass sich die Kontamination nicht weiter im Haus verbreitet.* (*Int3)*

Neben diesen Qualitätsverbesserungen in Form kontinuierlicher Messungen besteht die Möglichkeit, anhand einer App vereinfacht Ärzte zu kontaktieren (vgl. Int1). Insgesamt dienen Mobile-Health-Lösungen daher der Prozessoptimierung und Koordination der Akteure, womit eine höhere Effizienz erreicht wird (vgl. Int6).

### Handlungsempfehlung

**Abb. 3:**
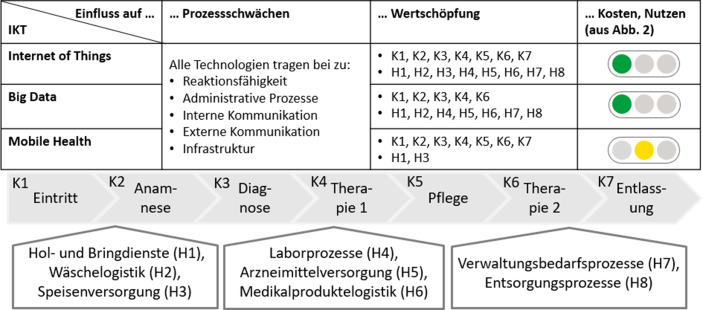
Einfluss von IoT, Big Data und Mobile Health (eigene Darstellung; Ergänzung von Abb. 2)

Zusammenfassend sind Investitionen in IoT-, Big-Data- und Mobile-Health-Lösungen wertschöpfungssteigernd, was aus Abb. 3 sowie den skizzierten Anwendungsmöglichkeiten ersichtlich wird.

Big-Data- und Mobile-Health-Lösungen stiften großen Nutzen, indem Diagnosen automatisiert oder Messungen von Wearables in das KIS eingelesen werden (vgl. Int3, Int1). Diese Anwendungsbeispiele führen zur effizienteren Behandlung, weshalb diese IKT die Reaktionsfähigkeit erhöhen (vgl. Int7). Daneben wird die Kommunikation durch die Kombination der drei beschriebenen IKT optimiert. Durch die Verwendung von Big Data und IoT werden Tätigkeiten vereinfacht oder automatisiert, indem zuvor nicht berücksichtigte Informationen zugänglich werden, was die Nachhaltigkeit dieser Lösungen in Bezug auf die Aspekte Konsistenz und Suffizienz unterstreicht (vgl. Int4). Erhalten Ärzte mehr Daten, erhöht sich deren Produktivität (vgl. Int1), sodass alle drei IKT die identifizierten Schwächen mindern und die Wertschöpfung gemäß These 3 erhöhen (vgl. Int3).

#### Handlungsempfehlung

IoT und Big Data besitzen langfristig einen positiven, nachhaltigen Einfluss auf sowohl die Breite als auch die Tiefe des Wertschöpfungsspektrums deutscher Krankenhäuser, weshalb deren Einsatz empfohlen wird. Mobile Health wirkt stattdessen mittelfristig und eingeschränkt vorteilhaft auf einige Teilbereiche, sodass die Implementierung bedingt empfohlen wird.

Allerdings existieren Hindernisse, welche die identifizierten Nutzenpotenziale einschränken. Durch die IKT-Nutzung kann es zu Versorgungsschwierigkeiten kommen, falls das Netzwerk nicht stabil oder die Geräte nicht funktionstüchtig sind, z. B. durch Fehlmessungen den Patienten verunsichern (vgl. Int6). Neben diesen technischen Aspekten existieren weitere exogene Hindernisse. 56 % der Onlinebefragten erkennen Grenzen in den derzeitigen Datenschutzrichtlinien, 66 % fehlende finanzielle Ressourcen. Der Staat, der für die Bereitstellung von Investitionsmitteln zuständig ist, stellt die Gelder für Innovationen sowie fachgerechtes IT-Personal nicht ausreichend zur Verfügung (vgl. Int1). Dennoch besteht in diesem Kontext die Möglichkeit, gemeinsame, alternative Finanzierungsoptionen mit dem Hersteller auszuhandeln. So installierte Hochtief im Evangelische Krankenhaus Hubertus in Berlin ein Blockheizkraftwerk, das durch die jährlichen Energieeinsparungen des Krankenhauses abbezahlt wird. Mit dieser Maßnahme gestaltet das Krankenhaus seine Entwicklung zum „Green Hospital“ der Zukunft (Hibbeler und Krüger-Brand 2013). Werden folglich diese Hindernisse überwunden, so ermöglichen die IKT positive Effekte auf die Krankenhäuser.

## Fazit

Die empirischen Erkenntnisse der Gap-Analyse bestätigen somit die These, dass bereits zum Erhebungszeitpunkt im Sommer 2019 Prozessschwachstellen in Krankenhäusern existierten. Ergebnis der Untersuchung sind Leistungs- und Nachhaltigkeitsdefizite in der internen wie externen Kommunikation, der Reaktionsfähigkeit, den internen Verwaltungsprozessen und der IT-Ausstattung. Diese Defizite werden nun durch die Corona-Situation erneut deutlich. Eine Lösungsmöglichkeit ist die verstärkte Nutzung neuer IKT in deutschen Krankenhäusern. Diese weist allerdings in der Betrachtung Verbesserungspotenziale auf, was zum Erhebungszeitpunkt mit der fehlenden Investitionsbereitschaft in digitale Entwicklungen erklärt wurde. Dennoch zeigt sich, dass jede der betrachteten IKT Anwendungsmöglichkeiten bietet, um die beschriebenen Leistungs- und Nachhaltigkeitsdefizite zu reduzieren. Der Einsatz von Big Data erhöht beispielsweise die Schnelligkeit in den Abläufen, indem Diagnosen automatisiert und verknüpft werden können. Zusammen mit Mobile-Health-Lösungen können Messergebnisse und andere Informationen ausgewertet werden, die in dem aktuellen Epidemie-Szenario die Zurückverfolgbarkeit eines Virus ermöglichen. Damit waren bereits 2019 Lösungsmöglichkeiten bekannt, die während der Corona-Situation enorme Vorteile bieten würden. Der Einsatz der beschriebenen Mobile-Health‑, IoT- und Big-Data-Lösungen hilft, den Herausforderungen der heutigen Zeit zu begegnen. So optimieren die diskutierten IKT die Behandlungsqualität des Patienten, indem Lösungen in Krisensituationen geschaffen werden, generieren Wettbewerbsvorteile, sodass der Erhalt der Krankenhäuser gesichert wird, und tragen zu nachhaltigeren Prozessabläufen bei, um Krankenhäuser ganzheitlich unter Berücksichtigung ökologischer, ökonomischer und sozialer Aspekte weiterzuentwickeln.

## Footnotes

1 Die Quellenangaben der Zitate beziehen sich auf die Nummer des Interviews und die Stelle im Transkript. Bei Verdichtungsmetaphern im Fließtext wird darauf zur besseren Lesbarkeit verzichtet. Die Benennungen der Interviews beziehen sich auf: Int1: Geschäftsführer eines großen, freigemeinnützigen Krankenhauses. Int2: Beauftragter für Medizinproduktesicherheit eines mittelgroßen, freigemeinnützigen Krankenhauses. Int3: Verwaltungsleiter eines großen, öffentlichen Krankenhauses. Int4: Verwaltungsleiter eines großen, öffentlichen Universitätskrankenhauses. Int5: Qualitätsmanagementbeauftragter eines mittleren, freigemeinnützigen Krankenhauses. Int6: Verwaltungsleiter eines kleinen, freigemeinnützigen Krankenhauses. Int7: Verwaltungsleiter eines kleinen, privaten Krankenhauses.
